# Humic Substance Photosensitized Degradation of Phthalate Esters Characterized by ^2^H and ^13^C Isotope Fractionation

**DOI:** 10.1021/acs.est.2c06783

**Published:** 2023-01-23

**Authors:** Ning Min, Jun Yao, Hao Li, Zhihui Chen, Wancheng Pang, Junjie Zhu, Steffen Kümmel, Thomas Schaefer, Hartmut Herrmann, Hans Hermann Richnow

**Affiliations:** † School of Water Resources and Environment and Research Center of Environmental Science and Engineering, Sino-Hungarian Joint Laboratory of Environmental Science and Health, China University of Geosciences (Beijing), 29 Xueyuan Road, Haidian District, 100083 Beijing, China; ‡ Department of Isotope Biogeochemistry, Helmholtz Centre for Environmental Research − UFZ, Permoserstraße 15, Leipzig 04318, Germany; § Atmospheric Chemistry Department (ACD), Leibniz Institute for Tropospheric Research (TROPOS), Permoserstraße 15, 04318 Leipzig, Germany; ∥ Isodetect Leipzig GmbH, Deutscher Platz 5b, Leipzig 04103, Germany

**Keywords:** humic substances, photosensitization, isotope fractionation, phthalate esters, radical reactions, photodegradation

## Abstract

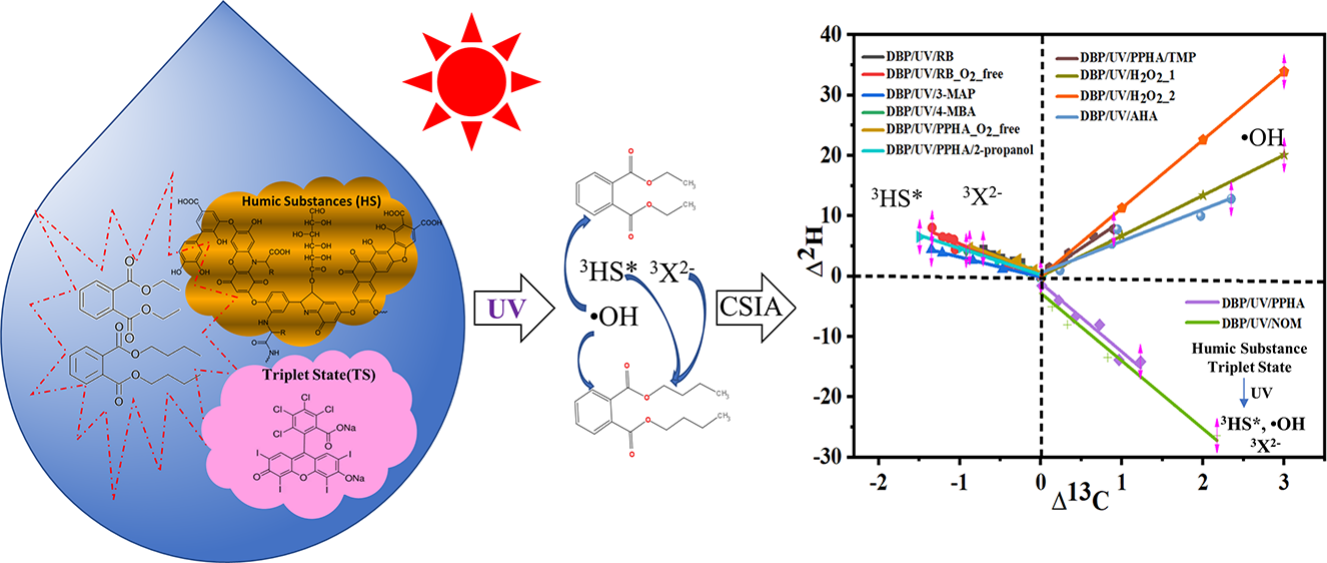

The photosensitized transformation of organic chemicals is an important degradation mechanism in natural surface waters, aerosols, and water films on surfaces. Dissolved organic matter including humic-like substances (HS), acting as photosensitizers that participate in electron transfer reactions, can generate a variety of reactive species, such as OH radicals and excited triplet-state HS (^3^HS*), which promote the degradation of organic compounds. We use phthalate esters, which are important contaminants found in wastewaters, landfills, soils, rivers, lakes, groundwaters, and mine tailings. We use phthalate esters as probes to study the reactivity of HS irradiated with artificial sunlight. Phthalate esters with different side-chain lengths were used as probes for elucidation of reaction mechanisms using ^2^H and ^13^C isotope fractionation. Reference experiments with the artificial photosensitizers 4,5,6,7-tetrachloro-2′,4′,5′,7′-tetraiodofluorescein (Rose Bengal), 3-methoxy-acetophenone (3-MAP), and 4-methoxybenzaldehyde (4-MBA) yielded characteristic fractionation factors (−4 ± 1, −4 ± 2, and −4 ± 1‰ for ^2^H; 0.7 ± 0.2, 1.0 ± 0.4, and 0.8 ± 0.2‰ for ^13^C), allowing interpretation of reaction mechanisms of humic substances with phthalate esters. The correlation of ^2^H and ^13^C fractions can be used diagnostically to determine photosensitized reactions in the environment and to differentiate among biodegradation, hydrolysis, and photosensitized HS reaction.

## Abstract 2

Multielement isotope fractionation was employed to probe the reaction mechanism of phthalate ester with different chain lengths in humic substance sensitized photodegradation.

## Introduction

Phthalate esters (synonym: phthalic acid esters) (PAEs), which are frequently used as plasticizers, are an important class of contaminants with medium persistence since they are reported to have potential carcinogenic, teratogenic, and mutagenic effects^1^ and have been classified as substances of very high concern. Additionally, they are suspected to interfere with the endocrine system, damage the immune system, interfere with the reproductive function of rodents and the regulation of the hypothalamic–pituitary–gonadal axis, and could have negative effects on children’s intellectual development.^2^ Based on their manifold use and high production quantity, PAEs are widespread in nature and appear in all environmental matrices such as water, air, sludge, soil, sediment, and mine tailings.^3−5^ Due to health and environmental concerns, the use of PAEs is getting restricted in the USA, the European Union, China, and elsewhere.^6^ In Europe, regulations prohibiting products containing certain phthalate esters have been implemented since 2000, and the United States Environmental Protection Agency (USEPA) identified dimethyl phthalic acid ester (DMP), diethyl phthalic acid ester (DEP), dibutyl phthalic acid ester (DBP), benzyl butyl phthalic acid ester, di-n-octyl phthalic acid ester (DOP), and bis(2-ethylhexyl) phthalic acid ester (DEHP) as priority pollutants. Major sinks of PAEs in the environment are anaerobic and aerobic biodegradation, hydrolysis, and photochemical degradation.^7,8^ Tracking the contribution of the different degradation mechanisms under environmental conditions is complex, but isotope fractionation may offer options to analyze degradation pathways (see below).

Generally, organic compounds can be degraded by direct and indirect photolysis.^9,10^ In direct photolysis, organic compounds directly absorb photons that induce structural transformations leading to a concentration decrease of the compound.^11^ In contrast, during indirect photolysis, a photosensitizer collects light, resulting in the formation of radical species, which can react with target compounds. Dissolved organic matter (DOM), which are ubiquitous in diverse aquatic environments, can be an important source of photochemically generated oxidants.^12^ Among others, humic substances (HS) are the main fractions of DOM, which can strongly absorb light and, upon excitation, can lead to the formation of free radical species ^3^HS*, ^•^OH, ^1^O_2_, O_2_^•–^, and e_aq_^–^, which can contribute to photodegradation reactions.^13−15^ In previous studies, ^•^OH and ^3^HS*, produced by HS, played a crucial role in the degradation of organic contaminants in sunlit surface waters.^13,16,17^ However, PAEs belong to pollutants that do not absorb light of the visible spectrum of the sun at the earth’s surface, resulting in a low degradation by direct photolysis.^18−20^ Therefore, only indirect photolysis can be a potentially relevant degradation process contributing to natural attenuation of PAEs in surface water bodies.

Until today, it is still an open question whether natural attenuation can be an effective and economic solution to treat PAE pollution, for example, in mine tailings.^3^ The degradation mechanisms of PAEs by sulfate radicals, OH radicals, hydrolysis,^21^ and biodegradation have been studied,^22^ but the degradation mechanism of PAEs induced by HS is currently unknown. Previous studies have reported degradation mechanisms of many organic pollutants by identifying potential intermediates of degradation processes by gas chromatography-mass spectrometry (GC/MS) or liquid chromatography-mass spectrometry (HPLC/MS).^23−29^ However, the identification of reaction mechanisms based on the analysis of degradation products can be uncertain, and thus, conclusions cannot reach a consensus.^22^ For studying the environmental behavior of organic pollutants, the identification of complex photosensitized mechanisms is urgent, which may be initiated by a variety of reactive radicals.

Compound-specific isotope analysis (CSIA) can be used as a complementary method to monitor changes of the isotopic composition of target compounds in a spatial or temporal context.^30,31^ In recent years, CSIA has been successfully used to evaluate transformation mechanisms and to estimate the degree of biodegradation of organic pollutants in the environment.^32,33^ When an organic pollutant is degraded, irreversible chemical bond changes usually result in isotope fractionation. Normal isotope effects are defined by an enrichment of the heavier isotopes (e.g., ^13^C) in the nonreacted residual fraction, which is accompanied by an enrichment of the lighter isotopes (e.g., ^12^C) in the formed transformation products.^34,35^ In contrast, in rare cases where the cleavage of heavy isotope substituted chemical bonds is favored, light isotopomers are enriched in the nonreactive fraction. These so-called inverse isotope effects are defined by an enrichment of heavy isotopes in the transformation products and an enrichment of light isotopes in the nonreacted fraction.^36,37^

The isotope fractionation can be used to further explore reaction mechanisms. The kinetic isotope effect (KIE) can be used to characterize the rate limitation of bond cleavage in the transition stage of a bond change reaction.^34^ Thus, the apparent KIE from experiments (AKIE) can provide information to describe the reaction mechanism of photosensitized reactions of phthalate esters based on the rate-limiting steps of a given reaction mechanism and the amplitude of the kinetic isotope effect, KIE.^38^

Within the present study, photosensitized degradation reactions of DEP and DBP employing humic substances (HS) were conducted to elucidate the underlying reaction mechanisms. Photosensitized reactions of these PAEs using Pahokee Peat humic acid (PPHA), Pahokee Peat fulvic acid (PPFA), Aldrich humic acid (AHA), and the photosensitizers Rose Bengal (RB), 3-methoxyacetophenone (3-MAP), and 4-methoxybenzaldehyde (4-MBA) as references were performed. Those reactions were investigated by CSIA to analyze the respective reaction mechanism. According to previous studies, ^•^OH radicals and the excited triplet state of HS (^3^HS*) are formed in the UV/PPHA system,^13^ which can be considered the model for humic substances. Rose Bengal, 3-MAP, and 4-MBA were used as model photosensitizing agents^39^ to produce reactive triple-state ^3^X^2–^ and subsequent ^1^O_2_ to compare the degradation mechanism of phthalate esters with different chain lengths (methyl to butyl) and HS to elucidate the degradation mechanism of PPHA acting as a photosensitizer.

The aims of this study are (I) to evaluate the degradation kinetics of PAEs in UV/HS, UV/RB, UV/3-MAP, and UV/4-MBA degradation experiments in the neutral condition; (II) to identify the transformation products using GC-MS and FT-ICR MS; (III) to study the ^13^C- and ^2^H isotope fractionation for characterization of the reaction processes in UV/RB, UV/RB/O_2_-free UV/3-MAP, UV/4-MBA, and UV/HS experiments; and (VI) to investigate the photosensitized reaction pathways of DEP and DBP in the systems of UV/HS and UV/triplet states.

## Experimental Section

### Chemicals

All chemicals were of analytical grade. Diethyl phthalate (DEP, diethyl benzene-1,2-dicarboxylate), dibutyl phthalate (DBP, dibutyl benzene-1,2-dicarboxylate), Rose Bengal (4,5,6,7-tetrachloro-2′,4′,5′,7′-tetraiodofluorescein, dye content 95%), 3-methoxyacetophenone (3-MAP), 4-methoxybenzaldehyde (4-MBA), 2-propanol-H_8_ (anhydrous), 2-propanol-D_8_ (anhydrous), hydrogen peroxide, dipotassium hydrogen phosphate (K_2_HPO_4_), potassium dihydrogen phosphate (KH_2_PO_4_), and 2,4,6-trimethyl-phenol (TMP) were purchased from Sigma-Aldrich. The solvents *n*-hexane and dichloromethane (HPLC grade) were supplied by the company Carl Roth. Pahokee Peat humic acid (PPHA; 1R103H-2), Pahokee Peat fulvic acid (PPFA; 2S103F), and Suwannee river natural organic matter (NOM, 2R101N) were purchased from the International Humic Substances Society (IHSS), and Aldrich humic acid (AHA) was derived from lignite and obtained from Sigma-Aldrich. Pure water (18 MΩ cm) was used to prepare the solutions using a Milli-Q system (Millipore, Billerica, MA).

### Experimental Setup for Photodegradation Reactions

Photodegradation experiments were conducted in a 300 mL Pyrex cylindrical flask with a quartz window using a 150 W xenon lamp as the light source (Type L2175, wavelength: 185–2000 nm, Hamamatsu Photonics K.K., Japan; Figure S2). Detailed information on the experimental setup can be found in the Supporting Information (SI).

### Photosensitized Degradation with H_2_O_2_

Photodegradation experiments of DEP and DBP in the UV/H_2_O_2_ experiment were conducted by filling the photoreactor with 300 mL of phosphate-buffered aqueous solution (pH 7, 10 mM) containing 0.8 mM DEP or 0.035 mM DBP, respectively. To minimize the headspace and to avoid volatility losses, the reactor was almost completely filled with the reaction solution and sealed. An initial molar ratio of 60:1 (H_2_O_2_: PAEs) was used for indirect photosensitized reactions with hydroxyl radicals (^•^OH). At different time points, aliquots of the reaction solution were taken using a syringe and the remaining PAEs were extracted by liquid–liquid extraction with *n*-hexane. The extracts were stored at −20 °C until analysis.

### Photodegradation with Humic Acids

PPHA, PPFA, NOM, and AHA-photosensitized degradation experiments of PAEs were conducted in a phosphate-buffered aqueous solution (pH 7.0, 10 mM). The initial concentration of humic acid was 0.4 g L^–1^. The concentrations of DEP and DBP were 0.8 and 0.035 mM, respectively. Changes in pH during the reaction were monitored using a pH meter (Mettler Delta 320, U.K.). Aliquots of the reaction mixture were removed at different time points, and the remaining PAEs were extracted by liquid–liquid extraction. DEP was extracted by adding 2 mL of DCM, which contained benzyl benzoate (500 mg L^–1^) as the internal standard. In contrast, DBP was extracted by adding 1 mL of *n*-hexane, which contained benzyl benzoate (50 mg L^–1^) as the internal standard. The different choice of extraction solvents depends on their extraction efficiency.^40^ A dark control experiment was conducted without addition of HS to the reaction mixture. A second control experiment was performed to investigate the effect of direct photolysis. Therefore, aqueous PAE solutions were irradiated in the absence of HS or H_2_O_2_, respectively. For the ^•^OH quenching trapping experiment, 2-propanol was added to the mixed solution of PPHA and PAEs, yielding an initial molar ratio of 60:1 (2-propanol: PAEs) before irradiation. For exploring the function of ^3^HS*, an oxygen-free experiment has been conducted in which the PPHA solution was purged with nitrogen to remove O_2_ for 1 h prior to the addition of PAEs. Purging with O_2_ or N_2_ resulted in negligible phthalic acid ester losses (for more information, see the SI). However, the phthalate ester concentration was determined before the start of irradiation in the experiments. To quench the reactions of ^3^HS* subsequently to form singlet oxygen and other reactive intermediates, 1 mM TMP was added to the reaction mixture.^13,41,42^

### Degradation Experiments Using Rose Bengal, 3-MAP, and 4-MBA as Photosensitizers

Experiments with Rose Bengal were performed to generate triplet-state and singlet oxygen. Therefore, 0.1 g of Rose Bengal was dissolved directly in 250 ml of phosphate-buffered solution, resulting in a final concentration of 0.39 mM. The solution was then adjusted to pH 7.0; the respective PAEs were added, and the solution was purged with oxygen to increase the O_2_ concentration or nitrogen to deplete the O_2_ concentration for 1 h before UV irradiation. For experiments with 3-MAP or 4-MBA, 5 mM stock solutions were prepared and stored at −4 °C until use. Aliquots of these stock solutions were added to DEP and DBP degradation experiments, yielding final concentrations of 0.4 and 0.2 mM, respectively.

### Analytical Methods

#### Concentration Analysis

Gas chromatography (7820A, Agilent Technologies) coupled with flame ionization detection (GC-FID) has been used to measure the concentration of target PAEs (DEP and DBP). The oven temperature program was initially 60 °C (held for 2 min), followed by a ramp of 10 °C min^–1^ to 290 °C and held for 2 min. All samples were injected in the split mode with the split ratio of 5:1, and the temperature of the injector was set at 250 °C.

#### Metabolite Analysis

Gas chromatography-mass spectrometry (GC-MS) was performed with GC (7890A, Agilent Technologies) connected with a Quadruple MS (5975C, Agilent Technologies). The GC was equipped with an HP-5 column (30 m × 0.25 mm × 0.25 μm; Agilent) for separation of analytes, and helium (1.5 mL min^–1^) was the carrier gas. GC-MS has been used to analyze nonpolar or weakly polar transformation products (TPs). All samples were injected in the split mode (5:1) into a split/splitless injector maintained at 250 °C. Additionally, Fourier transform ion cyclotron resonance mass spectrometry (FT-ICR MS) was used to analyze all possible TPs as described elsewhere.^43^

#### Isotope Analysis

Carbon and hydrogen isotopic compositions of PAEs were analyzed by a gas chromatograph-combustion-isotope ratio mass spectrometer (GC-C-IRMS), where a GC (7890A, Agilent Technologies) was connected through a GC-IsoLink and a ConFlo IV interface (Thermo Fisher Scientific, Germany) to a MAT 253 IRMS system (Thermo Fisher Scientific, Germany). Samples were injected in the split mode (5:1) for carbon or for hydrogen in the splitless mode into a split/splitless injector maintained at 250 °C. Samples were separated on a Zebron ZB-1 column (60 m × 0.32 mm × 1 μm; Phenomenex, Germany) with a constant carrier gas flow of 2 mL min^–1^ using the same temperature program as described for the analysis via GC analysis.

## Results and Discussion

### Photosensitization Experiments with Rose Bengal, 3-MAP, and 4-MBA as Models for Exited-State Reactions

Rose Bengal, 3-MAP, and 4-MBA were used to produce triplet states (^3^RB^2–^, ^3^3-MAP^2–^, ^3^4-MBA^2–^) that can either react with the phthalate esters or undergo an energy transfer with H_2_O or to ground-state molecular oxygen (O_2_) to form a singlet oxygen (^1^O_2_) during irradiation with UV light. The irradiation of RB^2–^ was performed in the presence or absence of O_2_; thus, the latter prevents the production of ^1^O_2_ as a reactive species. The DEP concentration in the dark control and direct photolysis experiments remained stable, indicating DEP is not degraded in control experiments (Figure S6). No DEP degradation was observed in the UV/RB and UV/3-MAP experiments, indicating that triplet-state reactions do not induce the degradation of DEP under the selected conditions (Figure S4). Further carbon and hydrogen isotope fractionation and transformation products were not detected in the control experiments and in the experiments DEP/UV, DEP/Rose Bengal/UV, DEP/3-MAP/UV, and DEP/4-MBA/UV in the presence of O_2_, which is consistent with the above results (Figure S7).

In contrast, the degradation of DBP was found in otherwise identical experiments with Rose Bengal, 3-MAP, and 4-MBA in the presence of UV light. The degradation of DBP followed first-order kinetics in the DBP/UV/triplet-state system (*R*^2^ > 0.96, Figure S4, Table 1). The rate constants (*k*) of degradation of DBP in the UV/RB, UV/3-MAP, and UV/4-MBA systems were 0.0168 ± 0.0058, 0.0244 ± 0.0009, and 0.0193 ± 0.0010 h^–1^, respectively. The results indicate that phthalate esters with longer side chains than ethyl groups undergo triplet-state reactions, suggesting that the reaction is dependent on the length of the alkyl side chain. In addition, the concentration remains stable in the control experiments in the dark and under direct photolysis (Figure S6), indicating that the degradation of DBP in the UV/RB, UV/3-MAP, and UV/4-MBA systems was caused by photosynthesized triplet-state reactions.

**Table 1:** Degradation Kinetic Parameters of DEP and DBP during Photochemical Oxidation^a^

	reaction system	photosensitizer concentration	dominant reactive species	*k* (h^–1^)	half-life (h)	*R*^2^
DEP	UV/PPHA/O_2_	0.4 g L^–1^	^•^OH, ^3^HS*	0.0077 ± 0.0012	129.87	0.99
	UV/PPHA/O_2__Free	0.4 g L^–1^	^3^HS*	n.d.	n.d.	n.d.
	UV/Rose Bengal/O_2_	0.39 mM	RB^2–^, ^1^O_2_	n.d.	n.d.	n.d.
	UV/3-MAP/O_2_	0.2 mM	3-MAP^2–^	n.d.	n.d.	n.d.
DBP	UV/NOM/O_2_	0.4 g L^–1^	^•^OH, ^3^HS*	0.0056 ± 0.0022	178.57	0.98
	UV/PPFA/O_2_	0.4 g L^–1^	^•^OH, ^3^HS*	0.0014 ± 0.0008	714.29	0.97
	UV/PPHA/O_2_	0.4 g L^–1^	^•^OH, ^3^HS*	0.0111 ± 0.0011	90.09	0.99
	UV/PPHA/O_2__Free	0.4 g L^–1^	^3^HS*	0.0036 ± 0.0007	277.78	0.98
	UV/PPHA/O_2_/TMP	0.4 g L^–1^	^•^OH	0.0043 ± 0.0011	232.56	0.97
	UV/PPHA/O_2_/2-propanol	0.4 g L^–1^	^3^HS*	0.0041 ± 0.0016	250	0.97
	UV/PPHA/O_2__Free/TMP	0.4 g L^–1^	n.d.	n.d.	n.d.	n.d.
	UV/AHA/O_2_	0.4 g L^–1^	^•^OH, ^3^HS*	0.0051 ± 0.0010	196.08	0.97
	UV/Rose Bengal	0.39 mM	RB^2–^, ^1^O_2_	0.0168 ± 0.0058	59.52	0.99
	UV/Rose Bengal/O_2__free	0.39 mM	RB^2–^	0.0166 ± 0.0015	60.24	0.96
	UV/3-MAP	0.2 mM	3-MAP^2–^	0.0244 ± 0.0009	40.98	0.96
	UV/4-MBA	0.2 mM	4-MBA^2–^	0.0193 ± 0.0010	51.81	0.98

a n.d., not detected as no degradation could be assessed.

### Photodegradation of DEP and DBP Using HS and UV Irradiation

PPHA, PPFA, and AHA were selected as models for the photosensitization of PAEs by a natural humic substance. The reaction was performed in the presence of O_2_, and both ^3^HS* and ^•^OH are expected. The results demonstrate that the degradation of DEP and DBP in this reaction can be described by first-order kinetics (*R*^2^ > 0.99, Table 1). Remarkably, the degradation rate constant (*k*) increased with the increasing chain length of the PAEs (*k*(DEP) = 0.0077 ± 0.0012 h^–1^; *k*(DBP) = 0.0111 ± 0.0011 h^–1^) for PPHA. The degradation rate constants (*k*) of DBP during photosensitizing reactions initiated by excited humic acids (*k* = 0.0051 ± 0.0010 h^–1^ for AHA, *k* = 0.0056 ± 0.0022 h^–1^ for NOM and *k* = 0.0111 ± 0.0011 h^–1^ for PPHA) were larger than those of fulvic acid (*k*(DBP) = 0.0014 ± 0.0008 h^–1^ for PPFA), suggesting that HAs were more efficient chromophores than FA, probably related to the larger aromatic moieties in HAs. In degradation experiments with DEP and DBP in the absence of O_2_, no degradation of DEP was observed, in contrast to a slow degradation of DBP (0.0036 ± 0.0007 h^–1^). In this experiment, the most important reactive species should be ^3^HS* as limited O_2_ concentration prevents the formation of ^1^O_2_. Thus, it can be concluded that ^3^HS* does not initiate the degradation of DEP in a UV/PPHA system without O_2_; however, ^3^HS* may initiate the transformation of DBP. These results were consistent with the experiments with artificial photosensitizers (RB, 3-MAP, and 4-MBA) as no degradation of DEP was observed (Figure S5, Figure S4, Table 1) but DBP can be degraded.

The DBP degradation experiments employing PPHA were conducted to elucidate the reaction mode. The O_2_-containing UV/PPHA experiment yielded a higher reaction rate (*k* = 0.0111 ± 0.0011 h^–1^, main reactive species are ^•^OH and ^3^HS*) in comparison to the O_2_-free setup (*k* = 0.0036 ± 0.0007 h^–1^), indicating that ^3^HS* was the major reacting major species. Oxygen plays an important role in the course of the reaction. The absence of oxygen would inhibit OH radical production. However, the degradation rate decreased significantly for both experimental setups when ^•^OH and ^3^HS* were quenched by the addition of 2-propanol or 2,4,6-trimethylphenol (TMP). TMP predominantly quenches triple-state reactions. A similar reaction kinetic (*k* = 0.0041 ± 0.0016 h^–1^ with the addition of 2-propanol and 0.0043 ± 0.0011 h^–1^ with the addition of TMP) was found. The similar reduction of the reaction kinetic in the quenching experiments indicates that ^•^OH and ^3^HS* have almost a similar contribution to the degradation of DBP in selected conditions. Typical transformation products with the hydroxylated benzene ring were not found in the experiments where the triplet state is dominant, supporting our interpretation (Table 1).

In the reaction of ^•^OH with 2-propanol-^2^H_8_, acetone is formed. In a previous study, deuterated 2-propanol-D_2_ was used to detect the ^•^OH.^44^ We used deuterated 2-propanol-D_8_ to detect the ^•^OH-induced conversion to acetone-D_6_ and to distinguish it from acetone produced by other reactions or originating as an impurity from laboratory air. Deuterated acetone-D_6_ was found in the system of PPHA under the irradiation of UV, indicating that ^•^OH was formed in the UV/HS/O_2_ experiment. The acetone concentration first increased with time and then decreased as the reaction progressed (Figure S8), indicating the acetone was transformed during the experiment, making quantitative assessment challenging. Nevertheless, it indicates that ^•^OH is a major reacting species formed by HS and degrades phthalate esters.

### Carbon and Hydrogen Isotope Fractionation during the Photodegradation of DEP and DBP

**Scheme 1:**
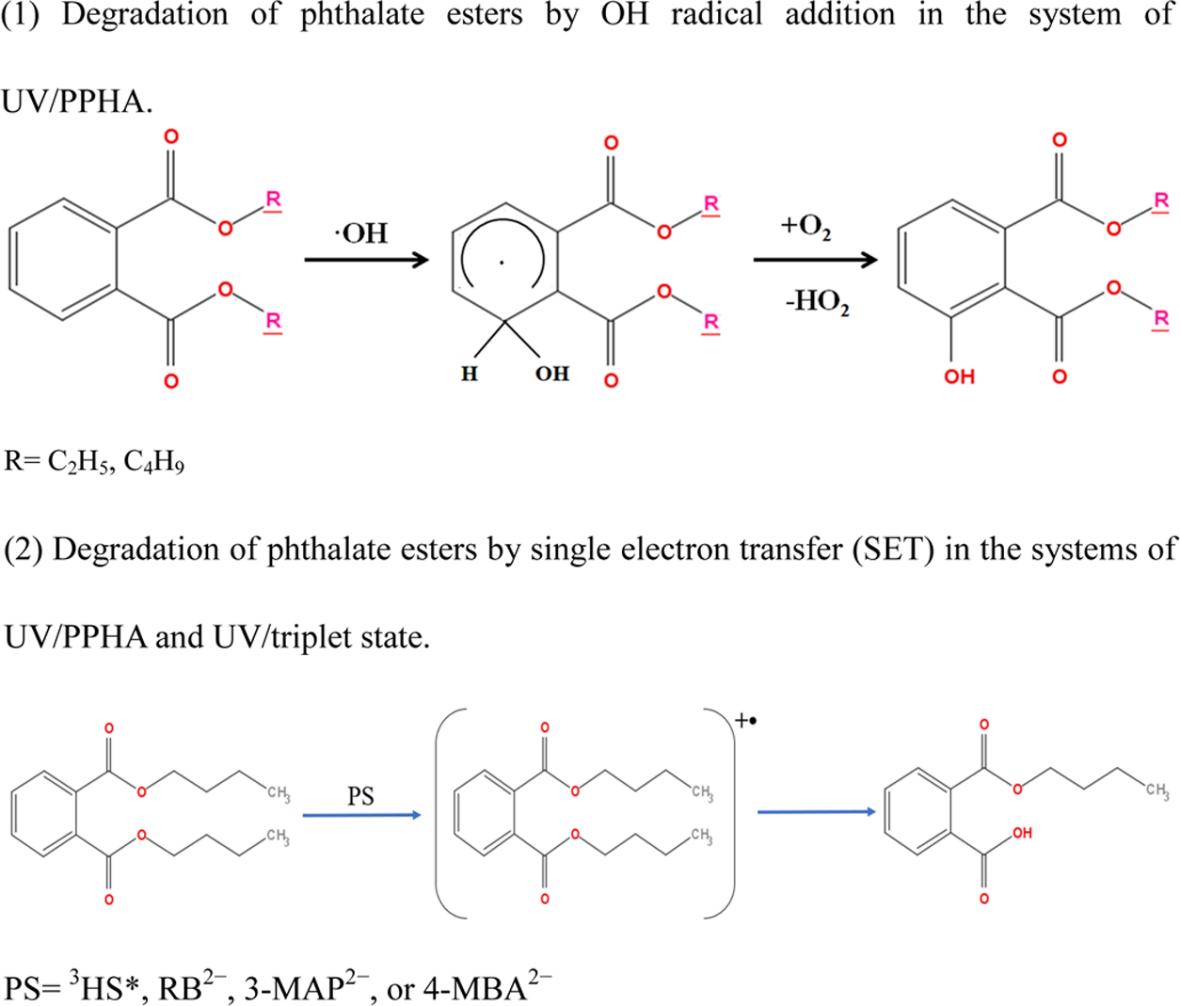
Degradation Pathways of DEP and DBP in Photosensitization Experiments

The carbon and hydrogen isotopic fractionation of DEP and DBP was studied for RB, 3-MAP, 4-MBA, and HS photosensitized reactions (Table 2). The reaction of DEP in the UV/PPHA system in the presence of O_2_ resulted in an isotopic fractionation for carbon of −1.8 ± 0.4‰ and for hydrogen of −9.0 ± 1‰, giving a Λ value of 3.2 ± 0.8. This corresponds to similar fractionation factors and Λ values observed in the H_2_O_2_/UV/DEP system (Λ = 2.4 ± 0.2). This result combined the transformation product, 3-hydroxydiethylphthalic acid ester, in the UV/PPHA/DEP system, indicating a dominance of the ^•^OH-induced reactions (Scheme 1(1)). This is in line with the finding that neither triplet-state reactions (^3^X^2–^) of 3RB^2–^, ^3^3-MAP^2–^, and ^3^4-MBA^2–^ nor ^3^HS* induced the degradation reaction and isotope fractionation of DEP (Table 2, Figure S9). The reaction of DBP in the UV/PPHA/TMP experiment in the presence of O_2_ resulted in an isotopic fractionation of −0.8 ± 0.3‰ for carbon and of −9.0 ± 2‰ for hydrogen, yielding a Λ value of 8.7 ± 2.5. This corresponds to similar fractionation factors and Λ values observed in the H_2_O_2_/UV/DPP experiment (Λ = 9.0 ± 2.3), indicating a dominance of the ^•^OH-induced reactions (Scheme 1(1)).

**Table 2:** Isotope Fractionation Parameters of DEP and DBP during Photochemical Oxidation^a^

	reaction system	concentration	εC (‰)	*R*^2^	εH (‰)	*R*^2^	Λ	
DEP	UV/PPHA/O_2_	0.4 g L^–1^	–1.8 ± 0.4	0.97	–9.0 ± 1	0.98	3.2 ± 0.8	this study
	UV/PPHA/O_2__Free	0.4 g L^–1^	n.d.	n.d.	n.d.	n.d.	n.d.	this study
	UV/Rose Bengal	0.39 mM	n.d.	n.d.	n.d.	n.d.	n.d.	this study
	UV/3-MAP	0.2 mM	n.d.	n.d.	n.d.	n.d.	n.d.	this study
	UV/H_2_O_2_	50 mM	–2.3 ± 0.4	0.99	-6.8 ± 1.3	0.99	2.4 ± 0.2	Zhang et al.^21^
DBP	UV/PPHA/O_2_	0.4 g L^–1^	-3.3 ± 1.0	0.98	28 ± 9	0.96	–11.4 ± 4.7	this study
	UV/AHA/O_2_	0.4 g L^–1^	-2.0 ± 0.9	0.99	-15 ± 5	0.91	5.3 ± 2.5	this study
	UV/NOM/O_2_	0.4 g L^–1^	-1.4 ±0.7	0.95	19 ± 3	0.90	–11.2 ± 5.7	this study
	UV/PPHA/O_2__Free	0.4 g L^–1^	0.9 ± 0.2	0.93	-12 ± 4	0.96	–6.2 ± 2.3	this study
	UV/PPHA/O_2_/2-propanol	0.4 g L^–1^	0.7 ± 0.2	0.99	-10 ± 4	0.97	–4.6 ± 1.5	this study
	UV/PPHA/O_2_/TMP	0.4 g L^–1^	–0.8 ± 0.3	0.97	–9 ± 2	0.97	8.7 ± 2.5	this study
	UV/PPHA/O_2__Free/TMP	0.4 g L^–1^	n.d.	n.d.	n.d.	n.d.	n.d.	this study
	UV/H_2_O_2_	2.15 mM	–0.9 ± 0.2	0.97	–9.3 ± 1.2	0.96	9.0 ± 2.3	Zhang et al^21^
	UV/Rose Bengal/O_2_	0.39 mM	0.7 ± 0.2	0.98	–4 ± 1	0.97	–5.8 ± 1.4	this study
	UV/Rose Bengal/O_2__free	0.39 mM	0.6 ± 0.2	0.97	–4 ± 1	0.99	–5.7 ± 2.5	this study
	UV/3-MAP	0.2 mM	1.0 ± 0.4	0.97	–4 ± 2	0.95	–4.8 ± 1.1	this study
	UV/4-MBA	0.2 mM	0.8 ± 0.2	0.93	–4 ± 1	0.99	–4.6 ± 1.4	this study

a Uncertainty given as 95% confidence interval. n.d., not detected, as no degradation could be assessed. n.a., not analyzed.

**Figure 1:**
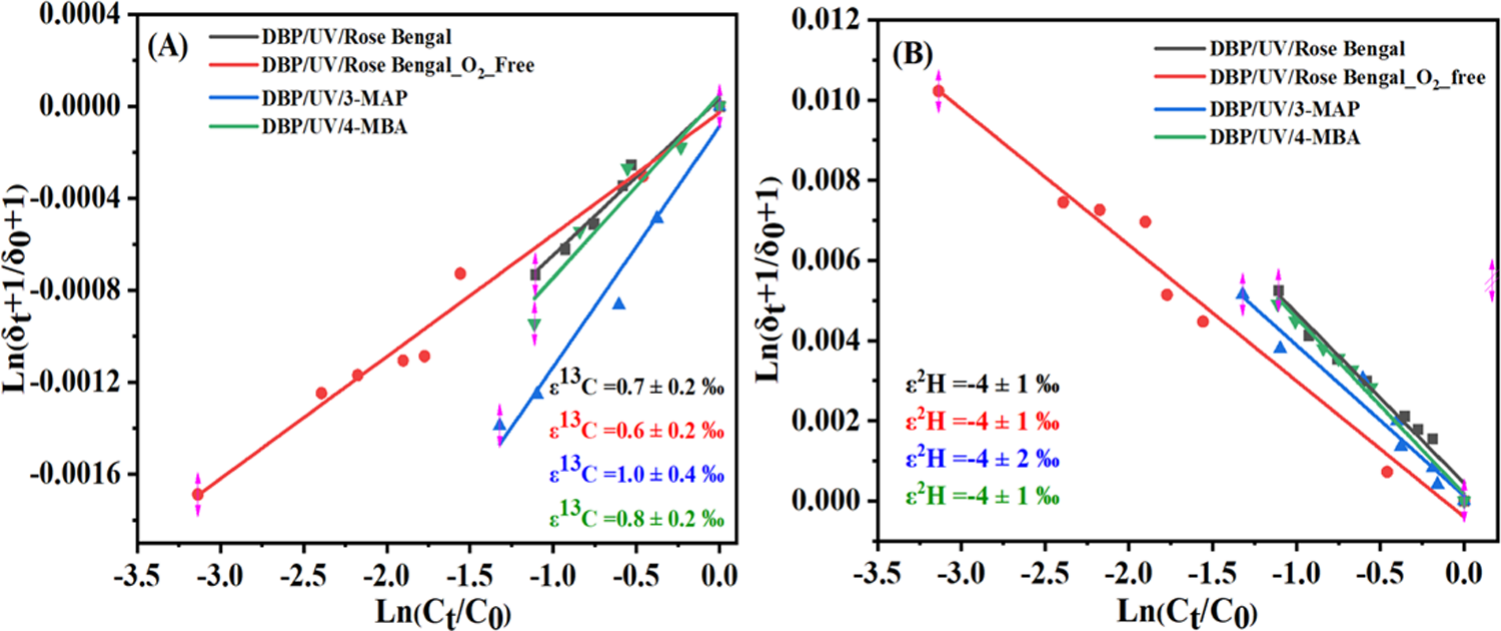
Rayleigh plots for carbon (A) and hydrogen (B) isotopic fractionation of DBP oxidation in the UV/Rose Bengal, UV/Rose Bengal_O_2__free, UV/3-MAP, and UV/4-MBA experiments. *C_t_*/*C*_0_ is the remaining fraction of substrate at time *t*. δ*_t_* and δ_0_ are the isotope compositions of substrate at times *t* and zero, respectively.

In contrast, the experiments with Bengal Rose in the presence and absence of O_2_, 3-MAP, and 4-MBA in the system of UV/DBP led to a small inverse carbon fractionation between 0.6 ± 0.2 and 1.0 ± 0.4‰ and normal hydrogen fractionation between −4 ± 1 and −4 ± 2‰ and with the characteristic Λ value between −5.8 ± 1.4 and −4.6 ± 1.4 (Table 2, Figure 1). Inverse carbon isotope effects have been observed previously in direct photolysis reactions, and magnetic isotope effect (MIE) has been cited as a source of uncommon inverse carbon isotope fractionation.^43,44^ In photochemical reactions, the mass-independent MIE originates from preferential selectivity for the triplet-state conversion of different magnetic isotopes. In direct photolysis reactions, the inverse C isotope fractionation of 2-chloroaniline photolysis was previously interpreted as evidence for increased bonding to ^13^C at the reactive atom in the rate-limiting step of the reaction.^45^ MIE during a direct photolysis reaction was hypothesized as a result of interactions in which nuclear spin and unpaired electrons of excited molecular radicals contribute to the lifetime of the intermediate species.^36,44^ Photosensitized ^1^O_2_ oxidation induced by Rose Bengal has led to inverse carbon isotope fractionation of tetrabromobisphenol A in water under simulated solar light irradiation, which may be similar to our reaction.^46^ However, tetrabromobisphenol A can adsorb light and might be degraded by direct photolysis. In contrast, the reference experiments with the photosensitizers such as Rose Bengal, 3-MAP, and 4-MBA catalyzing triplet-state reactions^37^ lead to inverse carbon fractionation in our reference experiments. The phthalate esters are transparent for wavelengths >280 nm, and the experiment with the filter indicates that triplet-state reactions are likely to be predominant and direct photolysis does not play a role.

Surprisingly, the reaction of DBP/UV/PPHA/O_2_ and DBP/UV/NOM/O_2_ yielded a normal carbon isotope effect (−3.3 ± 1.0‰ for PPHA and −1.4 ± 0.7‰ for NOM) but a pronounced inverse hydrogen isotope effect (28 ± 9‰ for PPHA and 19 ± 3‰ for NOM). A similar carbon isotope enrichment factor (−2.0 ± 0.9‰) has been found in the experiment with AHA in the presence of O_2_. AHA is derived from lignite with a relative high content of aromatic subunits, which may lead to photosensitized radical reactions different from peat humic substances and riverine organic material containing both humic and fulvic acids. The carbon and hydrogen fractionation of AHA-photosensitized reactions were in a similar range as expected for reaction with ^•^OH (Table 2). This is consistent with the experimental results of DBP photosensitized degradation experiments, suggesting an important contribution of ^•^OH-induced reactions. The results of the PPHA/UV/DBP experiment suggested a contribution of ^•^OH in the overall transformation; however, the mechanism of hydroxylation of the ring must be different as it led to a strong inverse ^2^H effect. Inverse ^2^H isotope fractionation indicates a reaction with a complex transition state due to an sp^2^ to sp^3^ hybridization at the reacting carbon, as observed in the reaction with alkylaromatic compounds.^21^ However, our reference reactions with ^•^OH showed normal ^2^H isotope effects, consistent with a previous work.^45^ Nevertheless, if the reaction was performed under oxygen-free conditions, the fractionation becomes similar to triplet-state reactions of HS with phthalate esters potentially by electron transfer (Scheme 1(2)).

**Figure 2:**
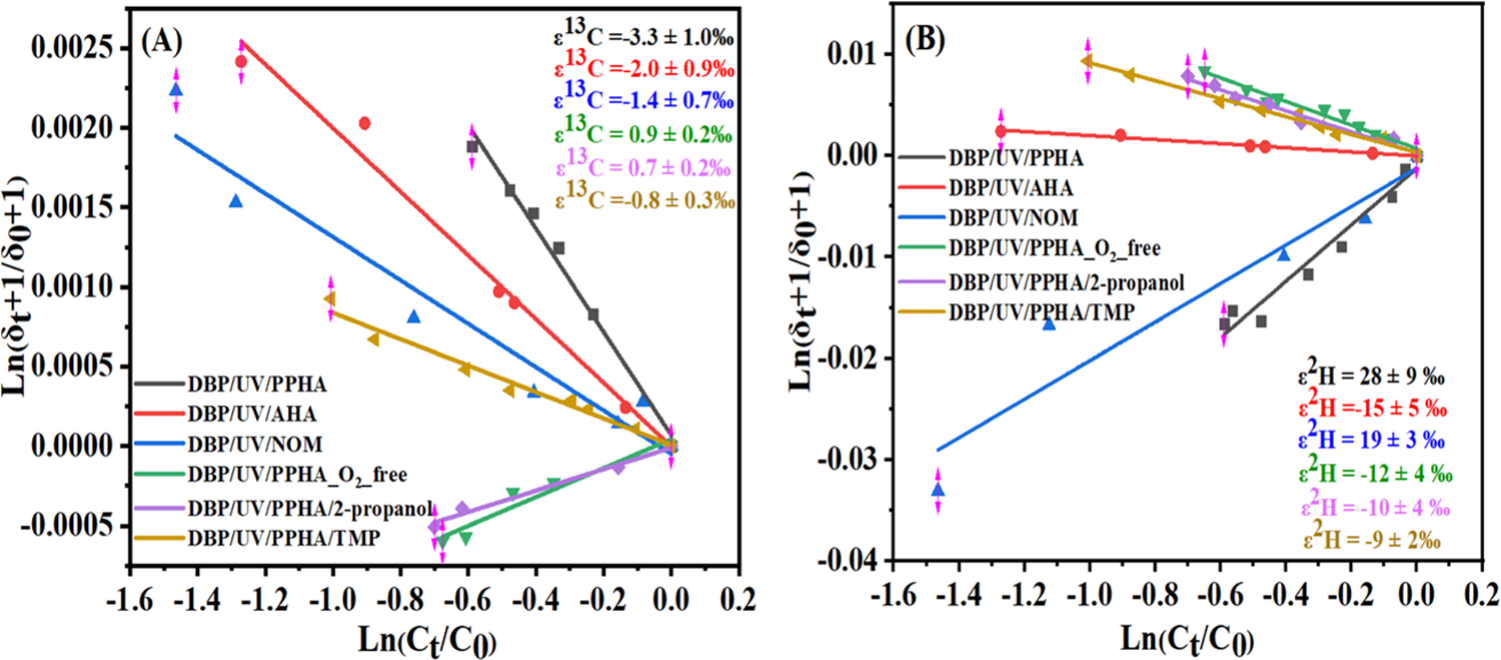
Rayleigh plots for carbon (A) and hydrogen (B) isotopic fractionation of DBP transformation in the UV/PPHA/O_2_, UV/PPHA/O_2_-free, UV/PPHA/O_2_/2-propanol, and UV/PPHA/O_2_/TMP experiments. *C_t_*/*C*_0_ is the remaining fraction of substrate at time *t*. δ*_t_* and δ_0_ are the isotope compositions of the substrate at times *t* and zero, respectively.

The ^•^OH quenching experiment with 2-propanol revealed a small inverse carbon isotopic fractionation (0.7 ± 0.2‰), which is similar to the triplet-state reactions (UV/RB, UV/3-MAP, and UV/4-MBA). In contrast, a normal hydrogen isotopic fractionation (−10 ± 4‰) was observed, which indicates that the reaction mechanism may have similarities to a triplet-state-induced reaction but with a different mechanism. Surprisingly, the hydrogen fractionation is similar to the ^•^OH reaction; however, ^•^OH cannot be quoted to give this pattern. Recall that experiment with PPHA in the absence of O_2_ (UV/PPHA/O_2__free) preventing generation of ^•^OH and quenching the ^•^OH reactions (UV/PPHA/2-propanol) slowed down the degradation of DBP. This shows the overall role of the triplet-state photosensitized reactions of HS. The carbon and hydrogen fractionations in the radicals quenched by TMP with DBP yield normal isotope effects of carbon (−0.8 ± 0.3‰) and hydrogen (−9 ± 2‰) and a Λ value of 8.7 ± 2.5. Zhang et al.^22^ observed similar normal isotope effects in ^•^OH reaction with DBP in photochemical experiments (UV/H_2_O_2_) (Figure 2). In summary, the characteristic pattern of carbon and hydrogen isotope fractionation allows one to determine the degradation pathways of DBP. A unique fractionation pattern has been found in the photosensitizer system compared with other mechanisms, for example, hydrolysis, microbial, and photodegradation (Table S1). In the few cultures studied, the biodegradation of DBP led to low normal ^13^C and undetectable ^2^H fractionation (Table S1), where hydrolysis of the ester bond is the first irreversible step. Chemical hydrolysis led to no or very low normal secondary ^2^H isotope effects, discussed in detail by Zhang and colleagues. The secondary ^2^H effect of alkaline hydrolysis is relatively pronounced for DMP, DEP, and DMP; however, it is always lower than observed in reactions with humic substances and triplet-state photosensitizers. Thus, the Λ values of hydrolysis are always lower than for humic substances, and triplet-state photosensitized reactions yield a different Λ due to inverse ^13^C fractionation. Only for the ^•^OH radical reaction with DMP and DEP are there some overlaps, and if the ^•^OH radical reaction becomes dominant in HS-catalyzed reactions, the correlation of ^2^H and ^13^C will lead to diagnostic uncertainty. Thus, in most cases, the Λ values may allow one to differentiate potentially environmentally relevant reactions such as biodegradation and acidic hydrolysis from humic acid-photosensitized reactions.

**Figure 3:**
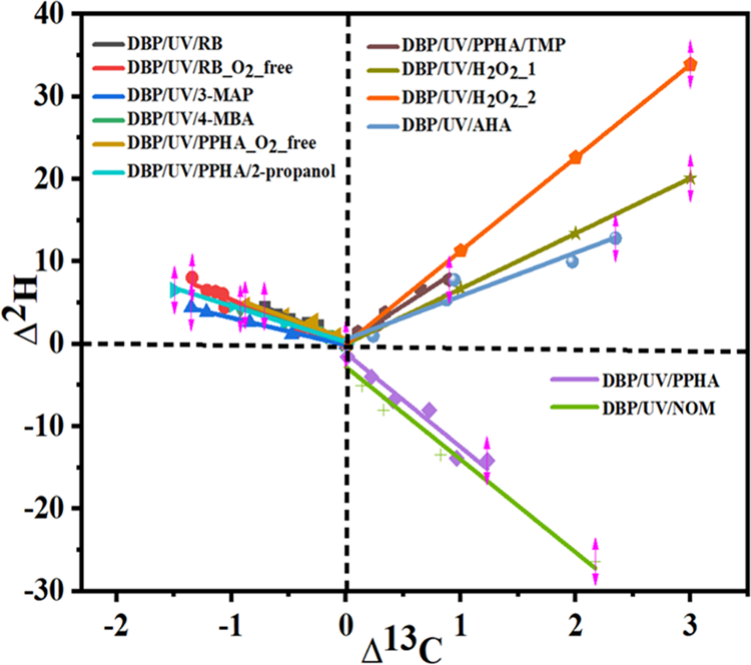
Two-dimensional (2D) plot for DBP degradation in the UV/PPHA/O_2_, UV/PPHA/O_2_-free, UV/PPHA/O_2_/2-propanol, UV/PPHA/O_2_/TMP, UV/PPHA/O_2_-free/TMP, UV/Rose Bengal, UV/Rose Bengal/O_2_-free, UV/3-MAP, and UV/4-MBA experiments.

In summary, although a number of reference and quenching experiments have been carried out with humic substances, the carbon and hydrogen isotope pattern cannot explain completely the inverse ^2^H fractionation. Most likely, both ^•^OH and triplet-state-induced reactions are involved; however, the precise mechanism of DBP transformation remains unresolved (Figure 3).

## Environmental Significance

The photodegradation promoted by photosensitizers could be an important degradation mechanism of phthalate esters and other organic contaminants in the environment. These reactions may be a significant factor governing organic pollutants’ attenuation because the reaction is not limited to low concentration compared to degradation by microbes using the contaminants as substrates for energy and growth. However, the contribution of photosensitizers to the natural attenuation of phthalate esters as well as their major degradation mechanism was missing due to lack of methods. Compound-specific isotope analysis (CSIA) may be used to identify the degradation pathways of phthalate esters in photosensitized reactions. The fractionation factors may be applied in future field studies to quantify reactions in the environment. The extent of ^13^C fractionation of triplet-state reactions (^3^TS^2–^) is relatively low, implying that the reaction progress is challenging to track very sensitively taking the typical analytical uncertainty of ±0.5‰ into account. The extent of ^2^H fractionation is larger compared to ^13^C fractionation, but also the analytical uncertainty of the H measurements ±5‰ is larger. This limits the quantification of quantifying HS photosynthesized reactions by ^2^H fractionation. However, isotope fractionation factors are mostly higher than hydrolysis and microbial degradation, enabling comparison of changes of the isotope pattern in field studies with respect to the various mechanisms.

Nevertheless, the mechanistic study using isotope fractionation may fill the gap for monitoring and identifying processes in the environment as the fractionation factors may be applied to quantify degradation in field studies using the Rayleigh concept.^47−49^ The isotope fractionation of multi-elements may be used diagnostically for identification of hydrolysis,^22^ microbial degradation,^21,50^ free radical addition reaction,^22^ as well as triplet-state reaction possible by single electron transfer (Table S1). CSIA has been applied to identify the degradation pathways of photoinduced reactions of humic substance and the triplet states of Rose Bengal, 3-MAP, and 4-MBA in the degradation of DEP and DBP. Thus, the application of CSIA could open new avenues for the assessment of these photoinduced pathways. Different degradation pathways can be elucidated by dual (δ^13^C and δ^2^H) isotope fractionation of DBP. The quenching experiment of ^•^OH radicals by 2-propanol in the presence of O_2_ in HS experiments may change the mechanism from H-atom abstraction to single electron transfer reaction, leading to inverse carbon isotope fractionation. Since the reactions associated with the triplet state do not induce DEP degradation, the triplet state may become relevant to phthalate ester degradation with longer side chains where the reaction occurs (Scheme 1(2)). The ^•^OH radical reactions lead to the addition of ^•^OH at the aromatic ring of phthalate esters, forming hydroxy phthalate esters. However, comparison of the ^2^H and ^13^C fractionation patterns of photosensitization of HS and reference experiments with the exited state indicate that several parallel reactions lead to the specific inverse C and normal H fractionation. The fractionation pattern can be used diagnostically to distinguish photochemical-induced reactions as well as biodegradation and hydrolysis.

The multi-element isotope fractionation model has been successfully developed to characterize the environmental fate and degradation pathways of phthalate ester degraded by sun-irritated humic material. Thus, the fractionation factors may be applied to analyze the photodegradation mechanism of phthalate ester using CSIA and can fill the gap in monitoring processes in the environment.

## Supporting Information Available

Details on experimental setup for photodegradation reactions, information on the kinetics of DEP and DBP transformation and control experiments, transformation of 2-propanol to characterize ^•^OH, and the identification of degradation product of PAEs by GC-MS and FTICT-MS (PDF)
