# Supplementary material for: Humic Substance Photosensitized Degradation of Phthalate Esters Characterized by 2H and 13C Isotope Fractionation
Source: Environ Sci Technol. 2023 Jan 23;57(5):1930–9. doi: 10.1021/acs.est.2c06783 (PMC9910037; doi:10.1021/acs.est.2c06783)
Supplement: Supplementary file 1 — es2c06783_si_001.pdf [file es2c06783_si_001.pdf]

Humic substance photosensitized degradation of phthalate esters characterized by ^2H and ^{13}C isotope fractionation and metabolite analysis

Ning Min[a,b], Jun Yao[a]*, Hao Li[a], Zhihun Chen[a], Wancheng Pang[a], Junjie Zhu[a], Steffen Kümmel[b], Thomas Schaefer[c], Hartmut Herrmann[c], Hans Hermann Richnow[a,b,d]*

[a]School of Water Resources and Environment and Research Center of Environmental Science and Engineering, Sino-Hungarian Joint Laboratory of Environmental Science and Health, China University of Geosciences (Beijing), 29 Xueyuan Road, Haidian District, 100083 Beijing, China

[b]Department of Isotope Biogeochemistry, Helmholtz Centre for Environmental Research – UFZ, Permoserstraße 15, 04318 Leipzig, Germany

[c]Atmospheric Chemistry Department (ACD), Leibniz Institute for Tropospheric Research (TROPOS), Permoserstraße 15, 04318 Leipzig, Germany.

[d]Isodetect Leipzig GmbH, Deutscher Platz 5b, Leipzig 04103, Germany

*Corresponding author: Jun Yao and Hans Hermann Richnow

E-mail: yaojun@cugb.edu.cn; hans.richnow@ufz.de

Phone: +86-10-82321958; +49(0)341-235-1212

Fax: +86-10-82321958; +49(0)341-235-2492

Content (13 pages)

1. Experimental setup for photodegradation reactions and light spectrum of the xenon lamp 3-4

2. Kinetics of the DEP and DBP transformation in the UV/HS and UV/triplet state experiments 5-6

3. Control experiments of PAE degradation 6-7

4. The concentration of acetone in the experiment with 2-propanol/ UV/HS 7-8

5. Stable isotope fractionation of DEP in experiment of UV/PPH 8-9

6. Identification of PAEs degradation products formed during photosensitization by GC-MS and FT-ICR MS 9-12

References 13

1. Experimental setup for photodegradation reactions and light spectrum of the xenon lamp

The photodegradation reactions of DEP and DBP were conducted in a 300-mL Pyrex cylindrical flask with quartz window (Figure S1). A 150 W xenon lamp (Type L2175, Hamamatsu Photonics, Japan) (Figure S2)[1] served as light source. To avoid direct photodegradation by UVC, a 280 nm cutoff filter was used to remove wavelengths shorter than 280 nm. The investigated PAEs dissolved in water showed no significant UV absorption at wavelengths longer than 280 nm (Figure S3)[2], thus no direct photolysis is expected. The temperature in the reactor was maintained at 20° C by circulating water through a water jacket connected with a cooler. The solution was magnetically stirred during the whole reaction process.

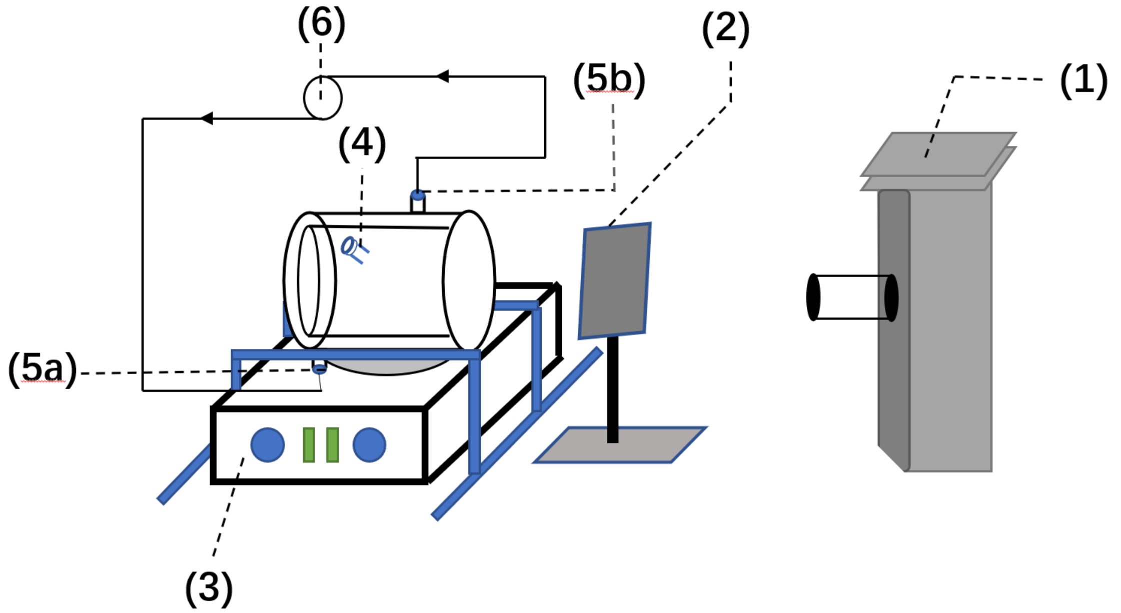

Figure S1. Experimental setup for photodegradation reactions. (1) 150 W xenon lamp; (2) 280 nm cutoff filter; (3) magnetic stirrer; (4) reactor with quartz window and sampling port; (5a) and (5b) water jacket with inlet and outlet for cooling water; (6) thermostat for cooling water.

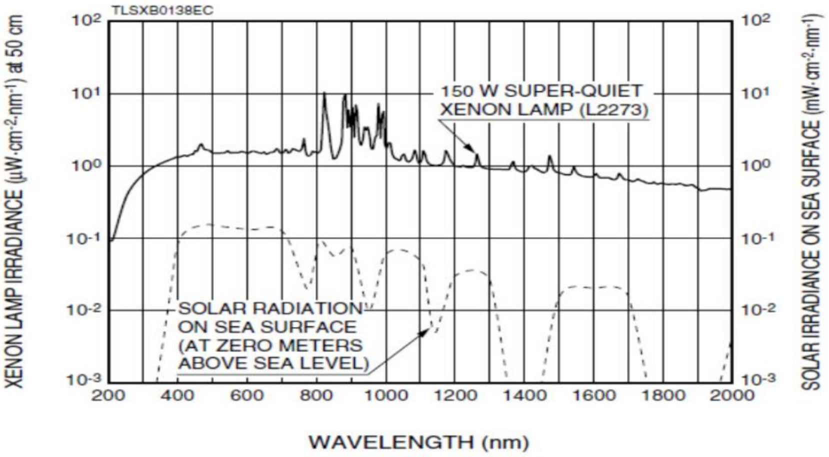

Figure S2. Light spectrum of the xenon lamp (Type L2175, Wavelength: 200-2000 nm, Hamamatsu Photonics, Japan).

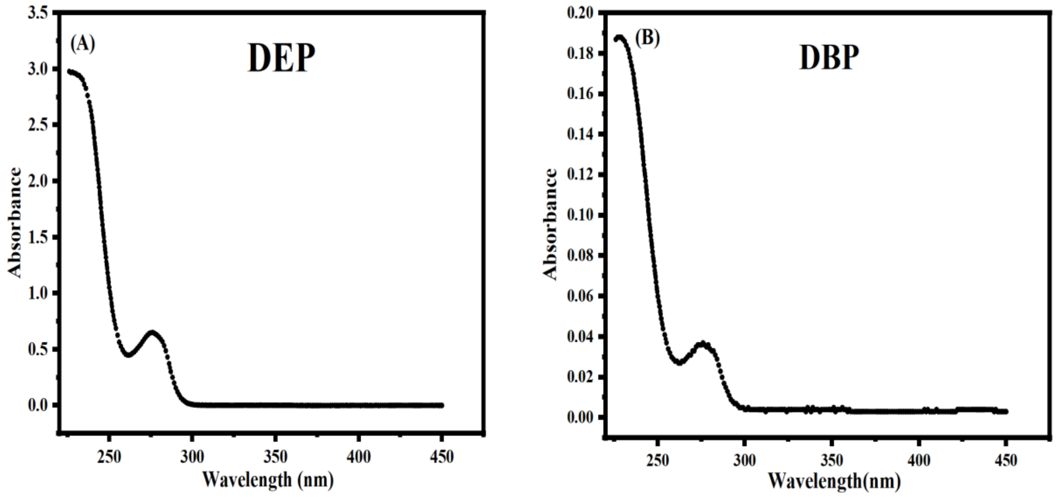

Figure S3. UV absorption spectrum of DEP (A) and DBP (B) in water at pH 7. The concentration of DEP and DEP are 0.8 mM and 0.035 mM, respectively. The absorption spectra were measured using a UV-VIS spectrometer (DR6000, Hach, USA)

2. Kinetics of the DEP and DBP transformation in the UV/HS and UV/triplet state experiments

In the UV/triplet state experiment, the concentration of DEP remained stable, indicating that neither Rose Bengal nor 3-MAP induced the degradation of DEP (Figure S4). In contrast, degradation was observed for DBP in the presence of Rose Bengal, 3-MAP and 4-MBA and the degradation can be described by a first-order kinetic (Figure S4).

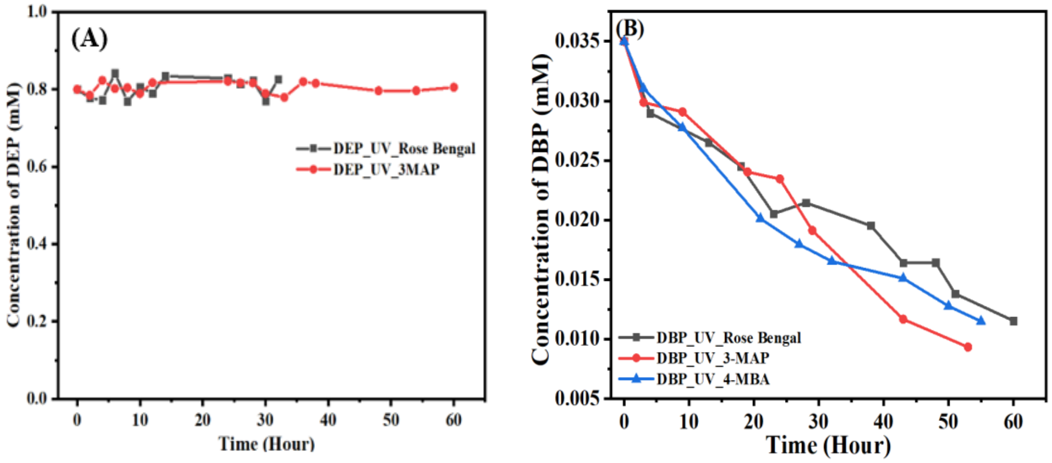

Figure S4. Degradation of DEP (A) and DBP (B) in the UV/Rose Bengal, UV/3-MAP and UV/4-MBA systems.

Both DEP and DBP degradation have been found in the experiment with UV/PPHA. The degradation can be described by a first-order kinetic (Figure S5).

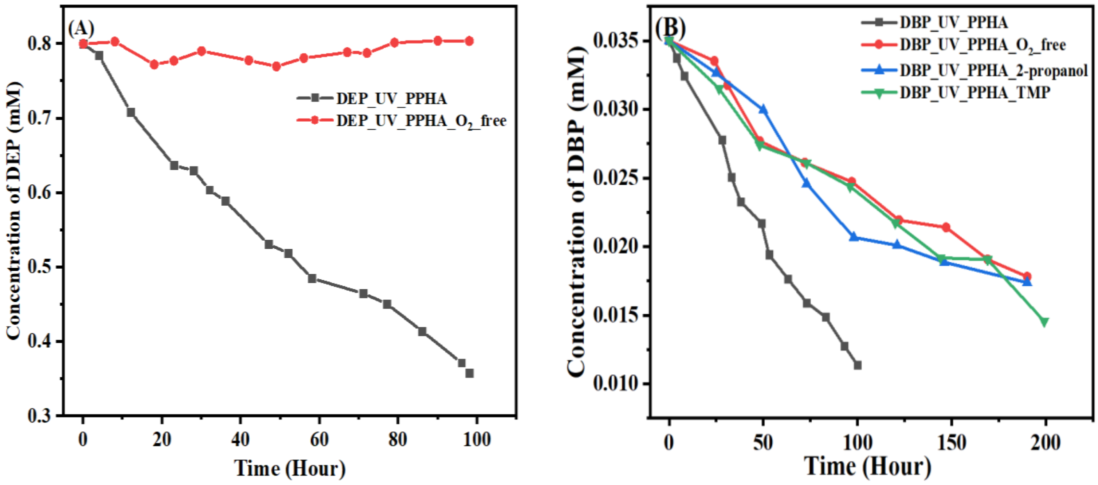

Figure S5. Degradation of DEP (A) in the UV/PPHA/O_2 and UV/PPHA/O_2-free experiments. DBP (B) in the UV/PPHA/O_2, UV/PPHA/O_2-free, UV/PPHA/O_2/ 2-propanol and

UV/PPHA/O_2/TMP experiments.

No apparent degradation of DEP was detected in the system of UV/PPHA/O_2-free experiment, which is consistent with the results in the UV/triplet state experiment. In addition, the degradation of DBP could be described by first-order kinetics in the experiment of UV/PPHA/O_2-free as well as in the quenching experiments using 2-propanol and TMP, which is in line with the results of in the UV/triplet state experiments supporting that a triplet state reaction dominate transformation. Optodes (SP-PSt3-NAU, PreSens GmbH) have been used to monitor the contraction of oxygen. The system has a detection limit of 15 ppb. The concentration of O_2 was below 60 ppb. The reactor was purged for 2 h and closed by a screw cap with a Teflon line septum. Sample were taken with a syringe and the hole in the septum was closed again with silicon glue immediately after sampling.

3. Control experiments of PAE degradation

Control experiments for the degradation of PAEs were conducted at pH 7. Two types of experiments were performed: I) UV irradiation but without catalyzer of triplet state reaction and PPHA; II) dark control experiments with triplet state reaction catalyst or PPHA. Under all experimental conditions, no significant decrease in PAEs concentration or significant fractionation of carbon or hydrogen isotopes of PAEs was observed (Figure S6; Figure S7).

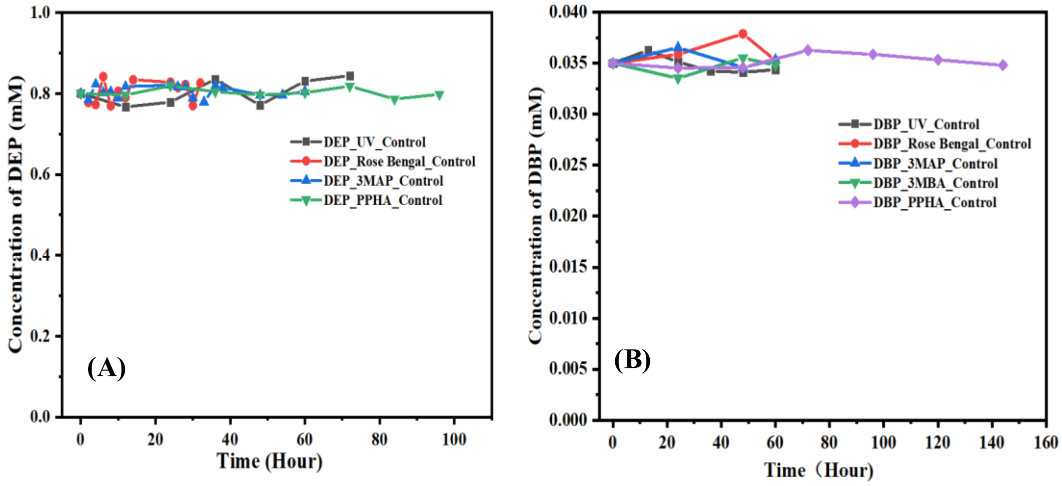

Figure S6. Concentration of remaining DEP (A) and DBP (B) fractions of control experiments, respectively (with photosensitizer of triplet state reaction, PPHA or UV irradiation only).

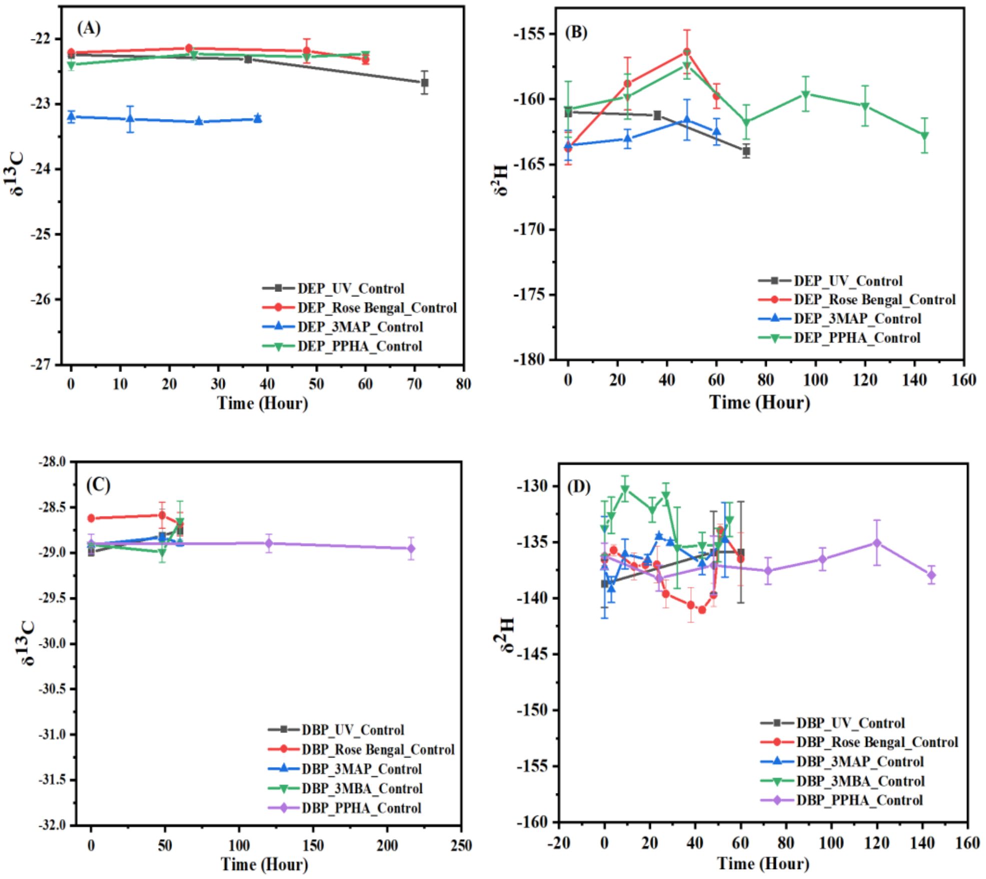

Figure S7. Stable isotope values (right panels) for $\delta^{13}C$ and $\delta^{2}H$ of DEP (A, B) and DBP (C, D) in control experiments (without photosensitizer of triplet state reaction or PPHA but UV irradiation, with photosensitizer of triplet state reaction or PPHA in the dark).

4. Experiments with isopropanol to prove OH radicals in photosensitization reactions

Experiments with 2-propanol and humic substance irradiated with artificial sunlight were conducted to detect •OH by the formation of acetone. In this reaction of ^{2}H-labelled 2-propanol with •OH, ^{2}H-labelled acetone is formed and can be clearly distinct from potential other acetone sources, such as reaction products HS or contamination from the laboratory. The results showed that acetone-$^{2}H_6$ was formed, indicating that •OH is an important radical in these experiments (Figure S8). The curve shows that acetone is rapidly formed and then further degraded.

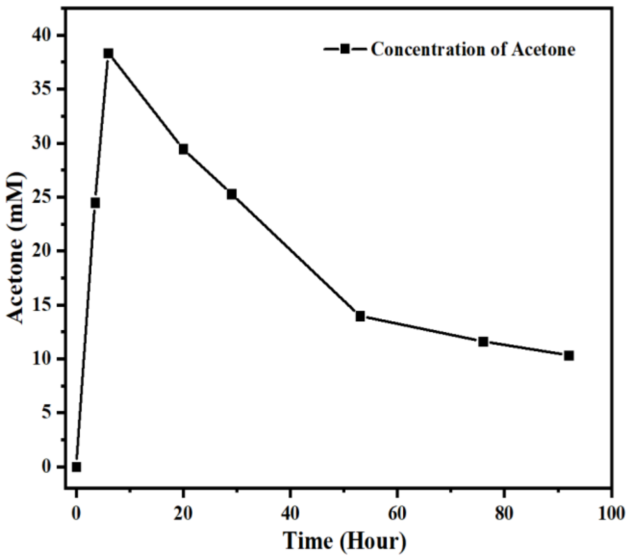

Figure S8. The concentration of acetone formed in the experiments with labelled 2-propanol and PPHA under the irradiation with artificial sunlight. [PPHA = 0.4 g L^{-1}, 2-propanol]$_0$ = 80 mM.

5. Stable isotope fractionation of DEP in experiment of UV/PPHA

A significant carbon and hydrogen isotopic fractionation of DEP was observed in the PPHA experiment irradiated with UV (>280 nm). Both the carbon and the hydrogen isotopic compositions of DEP became more negative, indicating a normal isotopic fractionation. The corresponding εC and εH were -1.8 ± 0.4‰ and -9.0 ± 1.3‰, respectively. The correlation of hydrogen and carbon isotopic fractionation (Λ =3.2 ± 0.8) gave statistically similar Λ values, compared to the Λ value observed for DEP degradation experiment with H_2O_2/UV (Λ = 2.4 ± 0.2) indicating the dominance of •OH in the photosensitization reaction.

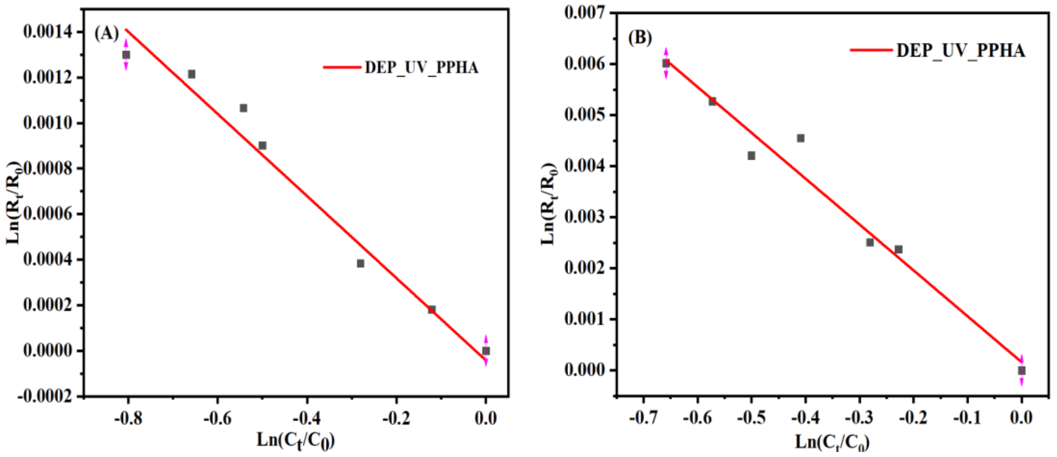

Figure S9. Rayleigh plots for carbon (A) and hydrogen (B) isotopic fractionation of DEP transformation in the oxygen-containing UV/PPHA system.

6. Identification of PAEs degradation products formed during photosensitization by GC-MS and FT-ICR MS

Potential transformation products were measured by GC-MS and FT-ICR MS[3]. Gas chromatography - mass spectrometry (GC-MS) has been used to analyzed non-polar or weakly polar transformation products (TPs). Additionally, Fourier-transform ion cyclotron resonance mass spectrometry (FT-ICR MS) was used to analyze all possible TPs in the reaction system. FT-ICR-MS has a very good sensitivity and mass resolution to organic acids which are expected to be found in the transformation products. In the reaction UV/PPHA/DEP, diethyl 3-hydroxyphthalate tentatively identified on the mass, was found as the predominant transformation product. Phthalic acid monobutyl ester has been identified as the main transformation product in the UV/triplet state model reactions using Rose Bengal, 3-MAP and 4-MBA as photosensitizer. In the UV/PPHA/O_2-free and UV/PPHA/2-propanol reaction systems, preferential attack of DBP side chains by the triplet state was observed, leading to phthalic acid monobutyl ester formation. In the reaction UV/PPHA/TMP, dibutyl 3-hydroxyphthalate were identified based on the mass as the main transformation product, due to •OH as the dominant radical species in this system. Both dibutyl 3-hydroxyphthalate and phthalic acid monobutyl ester have been found as the main metabolites in the UV/PPHA/DBP experiment in which •OH and triplet state reactions may take place in parallel.

Table SI Isotope fractionation of DMP, DEP and DBP during photochemical oxidation, hydrolysis and microbial degradation.

	Reaction system	pH	ε C (‰)	R^2	ε H (‰)	R^2	Λ	
DBP	Aerobic biodegradation by R. opacus DSM 43250	7	-1.1 ± 0.3	-	n.d.	-	n.d.	Zhang et al[4].
	Anoxic biodegradation by Bacillus sp. SASHJ	7	-0.5 ± 0.1	-	-	-	n.d.	Liu[47] et al.
	PS	7	-0.63 ± 0.07	0.989	-24.6 ± 1.8	0.996	35.3 ± 4.5	Zhang et al[21].
	Hydrolysis	2	-1.1 ± 0.1	-	n.d.	n.d.	n.d.	Zhang et al[4].
	Hydrolysis	7	-2.7 ± 0.4	-	n.d.	n.d.	n.d.	Zhang et al[4].
	Hydrolysis	10	-2.4 ± 0.1	-	−6 ± 1	0.99	1.9 ± 0.3	Zhang et al[4].
DEP	Hydrolysis	2	-2.6 ± 0.4	-	n.d.	0.99	n.d.	Zhang et al[4].
	Hydrolysis	7	-3.1 ± 0.4	-	-9 ± 2	0.99	2.5 ± 0.3	Zhang et al[4].
	Hydrolysis	10	-4.3 ± 0.3	-	-10 ± 1	0.99	1.9 ± 0.2	Zhang et al[4].
DMP	Hydrolysis	2	-3.4 ± 0.3	-	n.d.	0.99	n.d.	Zhang et al[4].
	Hydrolysis	7	-3.6 ± 0.3	-	-9 ± 1	0.99	2.5 ± 0.1	Zhang et al[4].
	Hydrolysis	10	-4.7 ± 0.2	-	-10 ± 1	0.99	3.0 ± 0.1	Zhang et al[4].
	UV/H_2O_2	7	−2.76 ± 0.25	0.99	−4.8 ± 0.5	0.99	2.0 ± 0.1	Zhang et al[2].
	PS	2	−2.09 ± 0.21	0.99	−23.9 ± 2.4	0.99	13.1 ± 1.4	Zhang et al[2].
	PS	7	−2.08 ± 0.10	0.99	−8.7 ± 1.2	0.99	4.8 ± 0.5	Zhang et al[2].

[a] Uncertainty given as 95% confidence interval. n.d.: not detected, as no degradation could be assessed. n.a.: not analyzed.

Table S2. Chemical formula of identified photoproducts.

Systems	Structure	Molecular formula	GC/MS	FT-ICR MS
DEP/UV/PPHA/ O_2	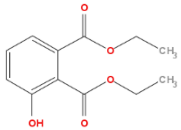	$C_{12}H_{14}O_5$	√	237.0768
DBP/UV/PPHA/O_2	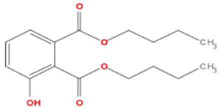	$C_{16}H_{24}O_5$	√	293.1394
	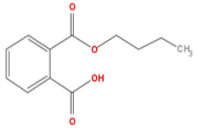	$C_{12}H_{14}O_4$	n.d.	221.0819
	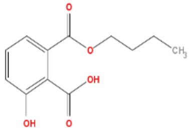	$C_{12}H_{14}O_5$	✓	237.0768
	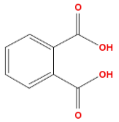	$C_8H_6O_4$	n.d.	165.0193
DBP/UV/PPHA/O_2_Free	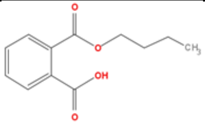	$C_{12}H_{14}O_4$	n.d.	221.0819
	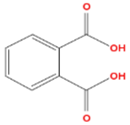	$C_8H_6O_4$	n.d.	165.0193

DBP/Rose Bengal/O_2	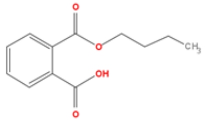	$C_{12}H_{14}O_4$	n.d.	221.0819
	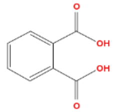	$C_8H_6O_4$	n.d.	165.0193
DBP/Rose Bengal/O_2_Free	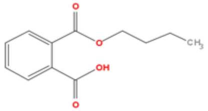	$C_{12}H_{14}O_4$	n.d.	221.0819
	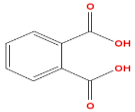	$C_8H_6O_4$	n.d.	165.0193
DBP/3-MAP/O_2	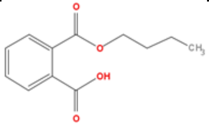	$C_{12}H_{14}O_4$	n.d.	221.0819
	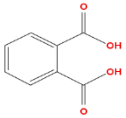	$C_8H_6O_4$	n.d.	165.0193

Reference

1. Zhang, N.; Schindelka, J.; Herrmann, H.; George, C.; Rosell, M.; Herrero-Martin, S.; Klan, P.; Richnow, H. H., Investigation of humic substance photosensitized reactions via carbon and hydrogen isotope fractionation. *Environ. Sci. Technol.* **2015,** *49*, (1), 233-42.
2. Zhang, D.; Wu, L.; Yao, J.; Herrmann, H.; Richnow, H.-H., Carbon and hydrogen isotope fractionation of phthalate esters during degradation by sulfate and hydroxyl radicals. *Chem. Eng. J.* **2018,** *347*, 111-118.
3. Wu, L.; Chladkova, B.; Lechtenfeld, O. J.; Lian, S.; Schindelka, J.; Herrmann, H.; Richnow, H. H., Characterizing chemical transformation of organophosphorus compounds by (13)C and (2)H stable isotope analysis. *Sci. Total. Environ.* **2018,** *615*, 20-28.
4. Zhang, D.; Wu, L.; Yao, J.; Vogt, C.; Richnow, H. H. Carbon and hydrogen isotopic fractionation during abiotic hydrolysis and aerobic biodegradation of phthalate esters. *Sci. Total. Environ.* **2019,** *660*, 559-566.
